# Experimentally Induced Anti-Myeloperoxidase Vasculitis Is Not Attenuated in Factor B or VISTA Deficient Mice

**DOI:** 10.1159/000521233

**Published:** 2021-11-30

**Authors:** Fernanda Flórez-Barrós, Simon J. Freeley, El Li Tham, Michael G. Robson

**Affiliations:** Faculty of Life Sciences and Medicine, Kings College London, London, UK

**Keywords:** Glomerulonephritis, Complement, V-type immunoglobulin domain-containing suppressor of T-cell activation, Inflammation, Vasculitis

## Abstract

**Background:**

Anti-neutrophil cytoplasmic antibody vasculitis is characterized by antibodies to myeloperoxidase or proteinase 3. Previous work in murine anti-myeloperoxidase vasculitis has shown a role for the alternative pathway complement component factor B and the anaphylatoxin C5a. However, mice deficient in properdin, which stabilizes the alternative pathway convertase, were not protected. V-Type immunoglobulin domain-containing suppressor of T-cell activation (VISTA)-deficient mice were protected in the nephrotoxic nephritis model but the role of VISTA in anti-myeloperoxidase vasculitis is unknown.

**Objectives:**

This study had 2 aims. First, we attempted to reproduce previous findings on the role of factor B in anti-myeloperoxidase vasculitis. Second, we examined the role of VISTA in this model, in order to see if the protection in the nephrotoxic nephritis model extended to anti-myeloperoxidase vasculitis.

**Methods:**

Anti-myeloperoxidase vasculitis was induced in wild type, factor B, or VISTA deficient mice. Disease was assessed by quantifying glomerular crescents and macrophages, in addition to albuminuria and serum creatinine.

**Results:**

When wild type and factor B deficient mice were compared, there were no differences in any of the histological or biochemical parameters of disease assessed. Similarly, when wild type or VISTA deficient mice were compared, there were no differences.

**Conclusions:**

Factor B deficient mice were not protected which is in contrast to previous studies. Therefore alternative pathway activation is not essential in this model, under the conditions used in this study. VISTA deficient mice were not protected, suggesting that therapies targeting VISTA may not be effective in vasculitis.

## Introduction

Anti-neutrophil cytoplasmic antibody vasculitis is a severe systemic disease which affects joints, lungs, kidneys, skin, and other tissues with most patients having autoantibodies against neutrophil and monocyte myeloperoxidase (MPO) or proteinase 3 (PR3) [1, 2]. Evidence that anti-MPO antibodies are pathogenic was provided by the observation that anti-MPO antibodies, raised by immunizing MPO-deficient mice with murine MPO, caused a focal necrotizing crescentic glomerulonephritis when injected into wild type mice [3].

Previous work has suggested that the alternative pathway is important as mice deficient in factor B but not C4 were protected [4]. In this model, C5 deficient mice are also protected, and treatment with an anti-C5 monoclonal antibody inhibited disease [5] providing evidence of a role for C5. Furthermore, MPO-deficient mice immunized with MPO and transplanted with bone marrow from C5a receptor deficient mice were protected from disease when compared with mice that received wild-type bone marrow, suggesting that the anaphylatoxin C5a is the key mediator [6]. This work has recently been extended with evidence of therapeutic efficacy for the C5a receptor antagonist CCX168 in mice in which the C5a receptor has been replaced with the human equivalent [7]. In addition, disease was exacerbated in mice defective in the second C5a receptor C5L2 [7].

Previous work from our group has confirmed that the C5 and C3 are important in pathogenesis but further showed that properdin was not required [8]. Properdin is a soluble protein that stabilizes the surface-bound C3 and C5 convertases of the alternative pathway increasing their half-life by up to 10-fold. This results in a greatly increased cleavage and activation of C3 and C5 by factor B [9]. Therefore, the lack of protection in properdin deficient mice was surprising given the previous data in factor B deficient mice. In another recent study, we demonstrated that V-type immunoglobulin domain-containing suppressor of T-cell activation (VISTA) deficiency protected from crescentic glomerulonephritis in the nephrotoxic nephritis model [10]. We showed that neutrophils were essential in nephrotoxic nephritis and that VISTA deficient mice had impaired neutrophil activation in response to immune complexes. The central role for neutrophils in anti-neutrophil cytoplasmic antibody vasculitis provided a rationale for studying the role of VISTA in the murine anti-MPO model.

In view of these recent findings, we decided to perform experiments, using the anti-myeloperoxidase model, to examine 2 distinct questions. First, we revisited the requirement for factor B, and second, we examined the role of VISTA.

## Materials and Methods

### Mice

Wild type C57BL/6 mice were purchased from Harlan (Bicester, Oxon, UK) or Charles River (Margate, UK) for experiments including factor B or VISTA deficient mice, respectively. Factor B deficient and VISTA deficient mice have been described [11, 12]. Mice were backcrossed to C57BL/6J for at least 10 generations. For experiments with factor B deficient mice, both male and female mice aged 8–10 weeks were used (the same proportion of male/female in each group). For experiments with VISTA deficient mice, female mice aged 6–8 weeks were used. Mice were age and weight matched for all experiments.

### Induction of anti-MPO Crescentic Glomerulonephritis

Anti-MPO antibody was raised in MPO-deficient mice as described and purified by protein G chromatography [13]. Glomerulonephritis was induced as described previously [13] with some modifications. Day 0 denoted the day that 2 mg of anti-MPO IgG was injected. In the experiments with factor B deficient mice, pegylated GCSF 30 μg (Neulasta; Amgen, Cambridge, UK) was given subcutaneously on day −8, −4, 0 and 4, and LPS 10 μg (*Escherichia coli* R515; Enzo Life Sciences, Exeter, UK − catalogue number ALX-581-007-L002) was given intraperitoneally on day 0 and 3. For experiments with VISTA deficient mice, GCSF 6 μg (Neulasta; Amgen, Cambridge) was given subcutaneously daily from day −4 to day +6. 50 μg/20 g of LPS (*Escherichia coli* R515; Enzo Life Sciences, UK − catalogue number ALX-581-007-L002) was given by intraperitoneal injection on day 0 and day 3. Spot urine was taken for urine albumin to creatinine ratio on day 6. In all experiments, mice were killed on day 7.

### Circulating Neutrophil Counts

Blood was taken from the saphenous vein on day −1. Total leukocyte counts were obtained after diluting whole blood in Turk's solution to lyse red cells (Merck, Nottingham, UK) and absolute numbers of neutrophils were calculated from percentage of neutrophils and total leukocyte numbers. Whole blood was stained with the appropriate antibodies with red cells were lysed using BD FACS Lysing Solution (BD Biosciences, Franklin Lakes, NY, USA) according to the manufacturer's instructions. A minimum of 10,000 events were collected per sample and data were analyzed using FlowJo software (Treestar, Ashland, OR, USA). Neutrophils were identified as Ly6G+ for the factor B experiment and as Ly6G+ CD11b+ for the VISTA experiment. For the factor B experiment anti-Ly6G; clone 1A8, BD Biosciences was used. For the VISTA experiment anti-Ly6G; clone 1A8, Biolegend and anti-CD11b; clone M1/70, eBiosciences were used. Flow cytometry was performed on a FACS Canto flow cytometer (factor B experiment) or a BD Fortessa flow cytometer (VISTA experiment) using FACSDiva software (BD Biosciences).

### Assessment of Disease

Kidney was fixed in Bouin's solution and stained with Periodic Acid Schiff. For immunofluorescence, an unlabeled primary antibody to CD68 (clone FA11, Serotec), and detection with Dylite 488 conjugated mouse anti-rat IgG (Jackson's Immunoresearch) was used. A minimum of 100 and 20 glomeruli per section were assessed on Periodic Acid Schiff stained and immunofluorescence sections, respectively. Serum creatinine was measured using liquid chromatography with tandem mass spectrometry (Pediatric clinical chemistry laboratory at Guy's and St Thomas' NHS Foundation Trust, London, UK) and urine albumin was measured by ELISA (Bethyl Laboratories, Montgomery, TX, USA). Urine creatinine was measured using a commercial creatinase assay (Diazyme, Dresden, Germany) with a 96 well plate reader and methodology based on the manufacturer's instructions, with a standard curve generated for all assays.

### Statistics

These were performed using Graphpad Prism version Graphpad Software, La Jolla, Laguna Hills, CA, USA). A Student's *t* test was used where 2 groups were compared. Some data were logarithmically transformed before analysis if the variances of the groups were significantly different.

## Results

### Factor B Deficient Mice in the anti-MPO Vasculitis Model

**Fig. 1:**
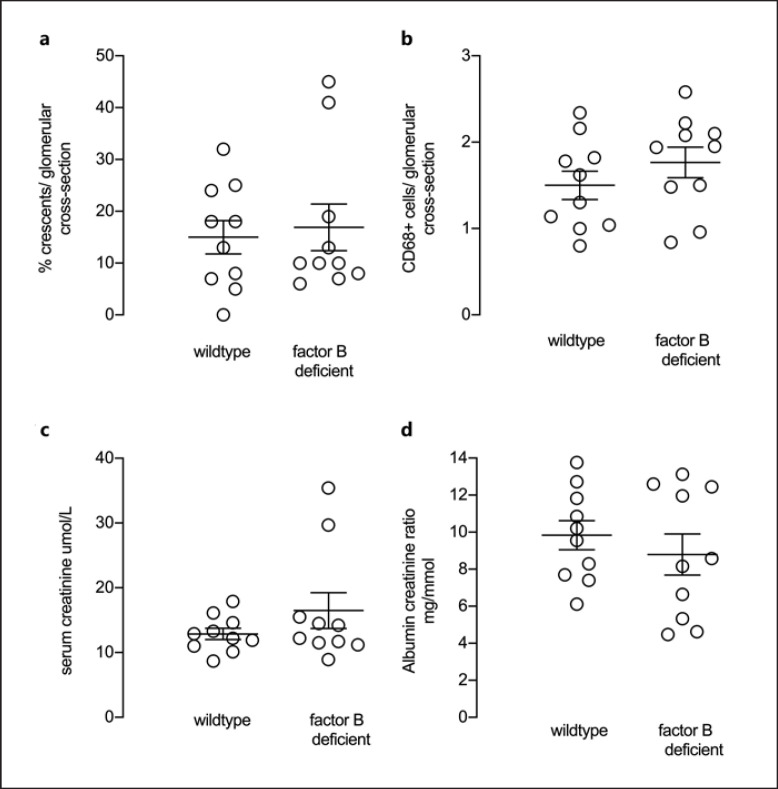
Histological and functional readouts of anti-MPO vasculitis in factor B deficient mice compared with wild types. **a, b** Histological readouts of glomerular crescents and glomerular CD68+ macrophages. **c, d** Functional readouts of serum creatinine and albuminuria. Each symbol represents a separate mouse. *N* = 10 per group. Error bars are mean ± SEM.

**Fig. 2:**
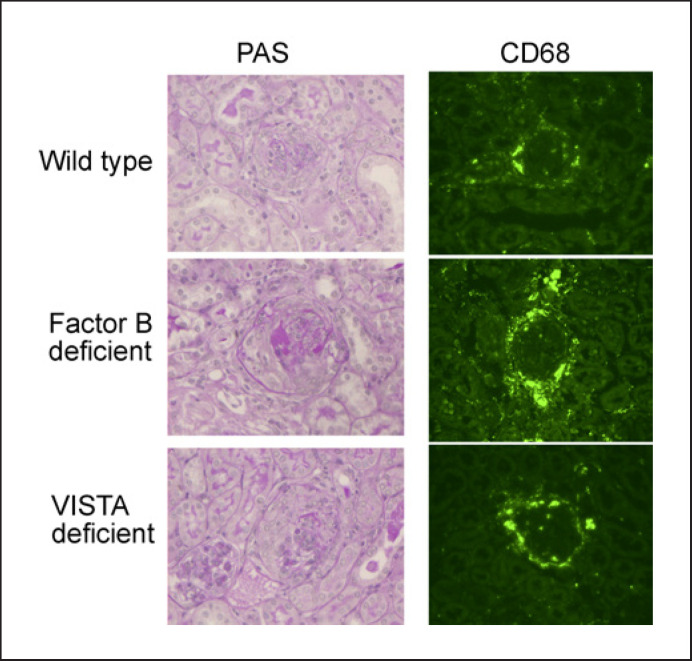
Representative renal histology showing PAS stained sections and immunofluorescence staining for CD68+ macrophages from mice with anti-MPO vasculitis. The strains shown include wild types, factor B and VISTA deficient mice. There were no differences between the groups and representative examples are shown. PAS, Periodic Acid Schiff.

We first induced crescentic glomerulonephritis in mice by injecting anti-MPO antibody and compared disease severity at day 7 in wild type and factor B deficient mice. Significant disease was induced in both groups with a mean percentage of crescents per glomerular cross section of 15 and 16.9 in wild type and factor B deficient mice, respectively. There was no difference in either crescents or glomerular CD68 positive macrophages between groups (Fig. 1a, b). Representative histology and immunofluorescence staining for CD68 are shown in Figure 2. We also assessed functional biochemical measures of disease. There were no differences in either serum creatinine or urine albumin creatinine ratio (Fig. 1c, d).

### VISTA Deficient Mice in the anti-MPO Vasculitis Model

**Fig. 3:**
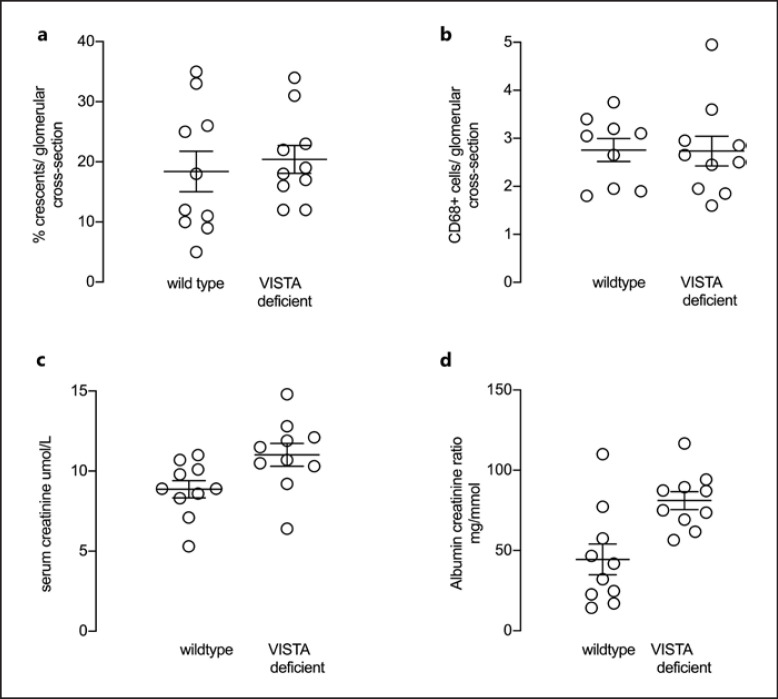
Histological and functional readouts of anti-MPO vasculitis in VISTA deficient mice compared with wild types. **a, b** Histological readouts of glomerular crescents and glomerular CD68+ macrophages. **c, d** Functional readouts of serum creatinine and albuminuria. Each symbol represents a separate mouse. *N* = 10 per group. Error bars are mean ± SEM.

Next, we first induced crescentic glomerulonephritis in mice by injecting anti-MPO antibody and compared disease severity at day 7 in wild type and VISTA deficient mice. The mean percentage of crescents per glomerular cross section was 18.4 and 20.4 in wild type and VISTA deficient mice respectively. There was no difference in either crescents or glomerular CD68 positive macrophages between groups (Fig. 3a, b). Representative histology and immunofluorescence staining for CD68 is shown in Figure 2. Furthermore, there were no significant differences in either serum creatinine or urine albumin creatinine ratio, although there was a trend toward an increase in proteinuria in VISTA deficient mice (Fig. 3c, d).

### Circulating Neutrophil Counts

**Fig. 4:**
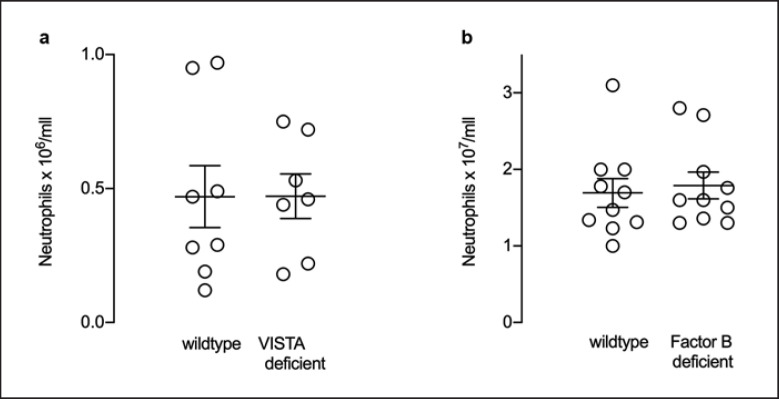
**a, b** Circulating peripheral blood neutrophils on the day before induction of anti-MPO vasculitis in VISTA deficient mice (Panel A, *N* = 7–8 per group) or factor B deficient mice (Panel B, *N* = 10 per group) compared with wild types. Each symbol represents a separate mouse. Error bars are mean ± SEM.

We measured circulating neutrophil counts on day −1 in order to exclude a difference in response to GCSF between strains. There were no differences when either VISTA deficient (Fig. 4a) or factor B deficient (Fig. 4b) mice were compared with wild types. Data on neutrophil counts were not obtained for the experiment in Figure 3. Therefore, the data in Figure 4a are from a different experiment with mice given GCSF according to an identical schedule. The neutrophil counts in both groups in the factor B experiment were higher than in the VISTA experiment, and this was due to the differences in the GCSF administration protocols.

## Discussion

In this brief report, we present 2 negative studies. First, we have failed to reproduce previous research showing an essential role for factor B in the anti-myeloperoxidase vasculitis model. Second, we have shown that VISTA deficiency mice are not protected from disease. We think it is important to publish these data. It is widely recognized that the scientific literature is biased toward positive results. Negative data are not as exciting but, if performed rigorously, just as significant. Furthermore, research requires resources and there are ethical issues regarding the use of animals. Therefore, negative data should be published as they will inform future work and help to avoid unnecessary repetition.

It is not clear why our data differ so significantly from a previous report showing a requirement for factor B in the development of anti-MPO vasculitis [4]. We confirmed the genotype of factor B deficient mice used in these experiments by PCR. We have previous published data showing lack of protection in this model for properdin deficient mice [8]. These previous results are consistent with our conclusion that the alternative pathway of complement is not important in anti-MPO vasculitis in our hands. The importance of the alternative pathway in a given experiment or model may depend on other factors including the severity of the disease induced. However, this is unlikely to be a major factor as the % crescents in wild-type mice was similar in our study compared to the previous report. Our model has some differences to other published models of anti-MPO vasculitis, including the use of LPS and GCSF and these are possible factors. Another variable to consider is the nature of the anti-MPO IgG used to induce disease. As this is polyclonal antibody, each batch that is used will necessarily be different.

We used GCSF in the experimental model as we have found that it is required for robust disease [13]. However, it was important to exclude a difference in response to GCSF between strains. If factor B or VISTA deficient mice had been more or less sensitive to GCSF then this could have affected the interpretation of results. However, the data presented showed similar circulating neutrophil counts in both groups of each experiment. This suggests that all strains responded equally to GCSF and that differences in sensitivity did not affect the results.

We have previously shown that C3 deficient mice were protected and confirmed work by others which showed protection in C5 deficient mice [8]. Furthermore, we found that C4 deficient mice were not protected which showed that the classical pathway of complement was not required for disease expression. Our previous experiments showing protection in C3 deficient mice were performed using an identical disease induction protocol to that used for factor B deficient mice. Our previous study in properdin deficient mice also suggests that the alternative pathway is not required and this also used the same disease induction protocol. Therefore, although we did not perform studies with C3 deficient and factor B deficient mice in the same experiment, the use of identical disease induction protocols means the findings can be interpreted together. In short, we have seen protection from disease in C3 and C5 deficient mice, with robust disease in factor B, properdin, and C4 deficient mice. The most plausible explanation for these findings is that both alternative and the classical pathway are required, but the absence of either pathway on its own may offer only insufficient protection due to their redundancy. This means that the presence of an intact classical or alternative pathway alone is sufficient to cause C3 activation.

Our results in VISTA deficient mice do not conflict with our previous published work in the nephrotoxic nephritis model [8]. A potential mechanism of protection in the nephrotoxic nephritis model was via inhibition of neutrophil activation by immune complexes. Glomerular immune complex deposition is not a feature of anti-MPO vasculitis. We were interested in examining the phenotype of VISTA deficient mice in anti-MPO vasculitis in case VISTA was important through other additional mechanisms. However, the lack of protection seen is consistent with a specific and dominant role for VISTA in immune complex mediated disease. In this regard, it is worth noting that a role for VISTA in collagen induced arthritis, another immune complex disease, has also been shown [14].

New therapies are needed for anti-neutrophil cytoplasmic antibody vasculitis in order to reduce both disease and treatment-related morbidity. Blockade of the C5a receptor has been shown to be effective, supporting the importance of the complement pathway in patients [15]. The data we present here suggest that specific inhibition of the alternative pathway may not be effective. We acknowledge that other groups have shown a role for the alternative pathway in murine model, and the role of factor B may vary with the experimental conditions used. We further show that therapies targeting VISTA may not be effective in anti-neutrophil cytoplasmic antibody vasculitis.

## Statement of Ethics

All animal experiments were performed under project licenses PPL 70/7448 and PPL P4D019509 which were approved by the King's College London Animal Welfare and Ethical Review Body and the UK Home Office.

## Conflict of Interest Statement

The authors have no competing financial interests to declare.

## Funding Sources

This work was supported by the Medical Research Council (MR/R004870/1), Genzyme Renal Innovations Program, and the Sir Jules Thorn Charitable Trust. We acknowledge financial support from the Department of Health via the National Institute for Health Research (NIHR) comprehensive Biomedical Research Centre (BRC) award to Guy's & St Thomas' NHS Foundation Trust in partnership with King's College London and King's College Hospital NHS Foundation Trust.

## Author Contributions

F.F.B., S.F., and E.T. performed experiments and analyzed data. M.R. conceived and supervised the research, analysed data and wrote the manuscript. All authors approved the final version of the manuscript.

## Data Availability Statement

Raw data will be made available in response to a reasonable request to the corresponding author from a qualified researcher.
